# Event and Comment

**Published:** 1905-10-15

**Authors:** 


THE
### DENTAL REGISTER.

Vol. LIX. October 15, 1905. No. 10.

### EVENT AND COMMENT.

### The Strained Editorial.

*Vide* recent dental journals: "Sorry is the lot" of those whose contract calls for a given amount of space each issue to be occupied by a *heavy* editorial. Heavy they usually are, and sometimes very "lumpy."

The above is a "Miscellaneous" comment by the Editor of the *Western Dental Journal*, and is characteristic of its author in that it contains much of truth and is plainly put, even though it may seem a triffle discourteous to us poor space writers who labor under the burden of a contract. There is no question but many editorials are heavy, and perhaps "lumpy," too; we confess our ignorance to this latter quality; our porridge is sometimes lumpy because it has not been stirred sufficiently while cooking. It may be that editorials should be stirred more while baking. We often think that we will surely stir the next batch better, but a month slips around so quickly and the printer is always calling for more copy and we are so busy that it is a wonder we do any stirring at all. Now we personally like the editorial even though it be heavy and "lumpy." The editorial, in such of our exchanges as condescend to print them, invariably get our first attention. We can hardly stop to get the wrappers off some of our exchanges to see what the editorial is about. We always want to know what Truman has to say and when Patterson does write an editorial we read it eagerly. We re-print two of his recent efforts in this issue. If it were not for the fear that we might make erroneous distinctions we should enjoy putting in print what we think of the editorials of our confrerers. We read them all with interest and we sincerely wish that this department of our journals could have more attention and appreciation.

**Editorial Qualities.**

The type of editorial, as exemplified in our professional journals, is rather difficult to define, because it is in most cases characteristic of the editor, or dictated by the business policy of the publishers. An editorial that is conventional should have the form of an official argument, or at least, be an expression of the mature judgment of one whose business it is to keep posted on the current events of his specialty. If we should make an attempt to judge the quality of the several journals which publish the literary output of our profession on this classic basis, we should be compelled to conclude that we have little or no editorial writing. It is perhaps well that we are not to be restricted by such a close definition. Many editors lack the capacity or inclination for grasping the general thought which occupies our professional world, unless it be made extraordinary obvious by some great discovery or insistent propaganda. Others may see it and lack the faculty of judicially estimating its importance, while some may realize the importance of these facts but be incapable of presenting them in an entertaining manner. There are few editors of dental journals that possess all these qualities of mind, therefore we have "heavy" or "lumpy" editorials, or what is worse, none at all. It can not be that there are no topics needing editorial comment. There never was a time of greater professional activity in the science, art, or literature of our calling, and it would seem that it was due the profession that the great passing events which are full of good as well as bad possibilities, should be brought into prominent notice by liberal and judicious editorial comment. Editors do not, nor should they be expected to know it all. They cannot dogmatically adjust all the vogarious notions of the clinical workers and the essayists, or even the scientific workers, yet they should keep the profession thinking properly about important professional matters. It may be necessary to have a department of humerous incidents, a record of deaths and marriages, a digest of current inventions of new processes and therapeutic formula, but these departments are of no permanent educational value unless edited into such form that they will cause men to ponder or analize them until their principles are assimilated. A brief editorial paragraph may be all that is necessary to present many important events, but the probabilities are that a long, and it may be a tedious or lumpy editorial, will best suit the editor in so expressing his opinion as to convey to the reader an adequate conception of the importance of events which may seem to be of little consequence by the average practitioner. Are we in danger of permitting our professional literature to degenerate into a sort of newspaper clipping bureau? The progress which our profession has made in the past has not been because we have learned to follow the suggestion given out by the wholly practical or technical dentist, we have become a profession because of the intelligent adjustment of our practice on a basis of principles tested and verified by scientific research.

**The Beginning of a New College Year.**

This is the season when the dental colleges open their doors for new classes of young men who seek places of usefulness in the world. The conditions under which they enter this year differ much from those in existence twenty-five years ago and probably will differ from those of twenty-five years hence. As we think of the young men who are entering these schools, we also find ourselves asking what there can be in the educational business that keeps a man twenty to thirty years in the business of teaching, especially when there is so little money to be gained by such an occupation. It is a laborious occupation, and the honors and money compensations never are adequate. It is possible that the egotism, which is so common an element of human character, is sufficient to make men believe that their message is of such great value to the world that it would be a sin to let it be hidden under a bushel, and that they are morally bound to see to it that it is most widely published. And how better can this be done than through the medium of the new set of dentists which are every year being turned out by the colleges? But every teacher is not an egotist, at least to the extent that he plans his life to publicly prove it. Pride or love of position may induce some men to work hard for small pay, because it insures position and honor, or preferment at least. But some of the oldest teachers are models of modesty and shun notoriety rather than seek it. Then it must be that they love the work for its own sake. It is true that the growth or development of the raw recruit from the high school to the graduate professional man is a vision of ever increasing pleasure. But all students do not develope either skill or learning, such of course, vex and annoy the teacher, but they are in the minority. The feeling that one is helping to shape a man's life into the possibilities of a great future of usefulness is a wholesome tonic, and keeps a man working long after he knows he ought to step aside or shift the heavy burden to others. In close connection with this idea is another inducement to continuous sacrifice and effort, and that is the intense devotion or consecration to duty which takes possession of one who knows that he has the confidence and respect of his students, and that he can exercise a function which he possess to the good of the student and the future betterment of the profession. It is surely these latter motives that possess a man who will work year after year in dental educational work, when he must know that by attending to his own business with the same interest and persistence, he would accumulate larger financial resources. As the richest man in the world has recently said, there are more gratifying things than the attainment of wealth. His idea of happiness is that one should strive to better humanity in general, which is the doctrine of the greatest teacher and benefactor of all times. And it is this idea and motive that keeps men faithful to the monotenous and unselfish occupation of teaching; and a man who is narrow or selfish should never undertake a calling which requires so largely devotion and self-sacrifice.

**Pyorrhea and Prophylaxis.**

We utilize the larger amount of this issue, to the exclusion of other matter, in presentiug two papers read before the last Michigan State Dental Association on Pyorrhea and Prophylaxis. We offer no apology for this use of our space because we know of no more important subjects for consideration; and we have never seen these subjects more clearly and forcibly presented. We trust our readers will take the time to carefully read both papers and the discussion. They are subjects which every dentist must be interested in whether he wants to be or not; and, he will find them most forcibly stated in these articles. Pyorrhea we must treat if we are to keep our patients, and we must treat it successfully if we are to keep their teeth. Prophylaxis seems to offer the most promise of accomplishing this desirable end, and every dentist must get to work at it soon if he wills to serve his patients adequately.

